# Clinical Risk Score for Predicting Recurrence Following a Cerebral Ischemic Event

**DOI:** 10.3389/fneur.2019.01106

**Published:** 2019-11-12

**Authors:** Durgesh Chaudhary, Vida Abedi, Jiang Li, Clemens M. Schirmer, Christoph J. Griessenauer, Ramin Zand

**Affiliations:** ^1^Neuroscience Institute, Geisinger Health System, Danville, PA, United States; ^2^Department of Molecular and Functional Genomics, Weis Center for Research, Geisinger Health System, Danville, PA, United States; ^3^Biocomplexity Institute, Virginia Tech, Blacksburg, VA, United States; ^4^Research Institute of Neurointervention, Paracelsus Medical University, Salzburg, Austria

**Keywords:** clinical risk scores, recurrent stroke risk, predictive modeling, ischemic stroke, predicting recurrence

## Abstract

**Introduction:** Recurrent stroke has a higher rate of death and disability. A number of risk scores have been developed to predict short-term and long-term risk of stroke following an initial episode of stroke or transient ischemic attack (TIA) with limited clinical utilities. In this paper, we review different risk score models and discuss their validity and clinical utilities.

**Methods:** The PubMed bibliographic database was searched for original research articles on the various risk scores for risk of stroke following an initial episode of stroke or TIA. The validation of the models was evaluated by examining the internal and external validation process as well as statistical methodology, the study power, as well as the accuracy and metrics such as sensitivity and specificity.

**Results:** Different risk score models have been derived from different study populations. Validation studies for these risk scores have produced conflicting results. Currently, ABCD^2^ score with diffusion weighted imaging (DWI) and Recurrence Risk Estimator at 90 days (RRE-90) are the two acceptable models for short-term risk prediction whereas Essen Stroke Risk Score (ESRS) and Stroke Prognosis Instrument-II (SPI-II) can be useful for prediction of long-term risk.

**Conclusion:** The clinical risk scores that currently exist for predicting short-term and long-term risk of recurrent cerebral ischemia are limited in their performance and clinical utilities. There is a need for a better predictive tool which can overcome the limitations of current predictive models. Application of machine learning methods in combination with electronic health records may provide platform for development of new-generation predictive tools.

## Introduction

Personalized medicine is dependent on data science and predictive analytics. This allows care providers to receive important alerts about potential events before they happen, and therefore make an informed decision. Predictive modeling of diseases that have a long-term effect can be crucial due to high individual and societal impact. Therefore, stroke as a leading cause of long-term disability is an essential target (1). It is vital and might be more practical to identify patients who are at a higher risk of recurrent cerebral ischemia. Recurrent stroke has a higher rate of death and disability and the long-term risk of recurrent ischemic stroke cannot be reliably assessed.

To date, a number of risk scores have been developed to predict short-term and long-term stroke recurrence after an initial episode of stroke or risk of stroke after transient ischemic attack (TIA). In this paper, we review different risk score models and discuss their validity and clinical utilities in the current clinical environment.

## Methods

The PubMed bibliographic database was searched for articles published on the various risk scores for recurrence of stroke. Search terms included: predictive scores stroke, predictive scores TIA, risk score stroke recurrence. We also explored the reference lists of the identified articles. All the relevant risk score models [e.g., ABCD, Essen Stroke Risk Score (ESRS)] were searched in the PubMed database. Studies were eligible for inclusion if they were original research articles in a peer-reviewed journal and were clinically applicable. Validation studies were required to have a follow-up of the reported cohort. The validation of the models was evaluated by examining the internal and external validation process. We also examined the statistical methodology, sample size, and the study power; as well as assessed the accuracy, and metrics such as sensitivity, specificity, and the receiver-operating-characteristic curve (ROC) (2). We paid attention to study population (general population- vs. hospital-based cohort and specialized center cohorts), study inclusion and exclusion criteria, and the outcome definition and measurement.

## Results

We identified 71 original articles describing, validating, or studying 16 risk scores to predict short-term or long-term recurrent cerebral ischemia (Tables 1, **5**). Each study was performed in different study populations and employed different methodology. The included studies had different inclusion and exclusion criteria. Many of the studies considered death as an outcome.

**Table 1:** Comparison of different predictive scoring models^*^.

**Name**	**Study type**	**Study population**	**Index event**	**Outcome**	**Validation studies**	**Clinical utility and potential limitations**
Stroke Prognosis Instrument (SPI-I)	Retrospective cohort (3)	142 patients, identified on a carotid ultrasound roster	First-time carotid TIA or minor stroke	Stroke or death within 2 years	2/2	Limited. Overestimates in low-risk patients; followed by SPI-II
SPI-II	Prospective cohort (4)	525 patients, WEST cohort	First-time carotid territory TIA or non-disabling stroke	Stroke or death within 2 years	4/4	Limited for early prediction. Overestimates in low-risk patients; carotid territory TIA or minor stroke only, Overall limited performance
Essen Stroke Risk Score (ESRS)	Prospective cohort (5)	6,431 patients, ischemic stroke subgroup of CAPRIE trial	Ischemic stroke	Recurrent stroke within 1 year	7/9	Limited for early prediction. Perform better for combined recurrent stroke and death, Overall limited performance
Recurrence Risk Estimator at 90 days (RRE-90)	Retrospective cohort (6)	1,458 patients, consecutive patients with ischemic stroke admitted to a single center	Ischemic stroke	Recurrent stroke within 90-days	4/4	Web-based prognostic tool. Limited use as it requires both accurate subtyping and neuroradiological assessment
Dutch TIA	Prospective cohort (7)	997 patients with TIA and 2,130 patients with minor stroke	TIA or minor stroke	Fatal or non-fatal stroke and for MI, stroke, or vascular death at 2 years	0/2	Overestimate the risk, Lacking validation
LiLAC	Prospective cohort (8)	2,473 participants of the Dutch TIA Trial	TIA or minor stroke	All-cause mortality and the composite event of death from all vascular causes, non-fatal stroke, and non-fatal MI at 10 years	0/1	Lacking validation
Hankey Score	Prospective cohort (9)	469 patients	TIA	Stroke; coronary event; and stroke, MI or vascular death within 5 years	3/4	Poor performance in validation studies involves complex equations limiting its clinical utility
California Risk Score	Retrospective cohort (10)	1,707 patients	TIA	Recurrent stroke at 90 days	2/2	Followed by ABCD^2^
ABCD	Prospective (population-based) cohort (11)	209 Patients	Probable or definite TIA	Stroke within 7 days	9/12	Followed by ABCD^2^
ABCD^2^	Derived from California risk score and ABCD and validated on the derivation cohort of these scores (12)	1,707 and 209 patients	TIA	Stroke at 2, 7, and 90 days	20/32	Most widely used for prediction of short-term risk of stroke, Questionable predictive value might underestimate the risk

* *Predictive scoring models derived from ABCD/ABCD^2^ are described in Table 5*.

### Stroke Prognosis Instrument (SPI)

SPI-I (Stroke Prognosis Instrument-I) was developed in 1991 as a prognostic system for patients with a carotid transient ischemic attack (TIA) or minor stroke to estimate the risk of stroke or death within 2 years (3).

The initial prognostic system consisted of three factors- age > 65 years (3 points), diabetes mellitus (3 points), and hypertension (2 points); and three risk groups- risk group I (0 points), II (1–5 points), and III (6–8 points). The development of the system was done in a cohort of 142 patients (Yale cohort) (3) in which the rate of stroke or death was found to be 2, 31, and 54% in the three risk groups, respectively (*P* < 0.0001). The initial system was testeds in another independent cohort of 330 patients (Canadian cohort) (3) in which the outcome rates were found to be 12, 21, and 31% in the three groups, respectively (*P* = 0.04). It was concluded that the initial system did not work in the second cohort as well as it did in the initial cohort. The high outcome rate in the first risk group (group I) in the Canadian cohort was of concern. Also, the outcome rates in the risk groups II and III were comparatively less in the Canadian cohort than in the initial cohort. To overcome these differences, the addition of coronary heart disease (1 point) and the distinction between TIA and stroke at baseline event (2 points) to the initial system led to the final SPI-I as given in Table 2.

**Table 2:** Parameters in SPI-I and SPI-II.

**Score**	**SPI-I**	**SPI-II**
Risk factors (points)	Age > 65 years (3) Diabetes mellitus (3) Hypertension (2) Coronary heart disease (1) Distinction between stroke and TIA at baseline event (2)	Age > 70 years (2) Diabetes mellitus (3) Hypertension (1) Coronary heart disease (1) Distinction between stroke and TIA at baseline event (2) Congestive heart failure (3) Prior stroke (3)
Risk groups	Group I: 0–2 points Group II: 3–6 points Group III: 7–11 points	Group I: 0–3 points Group II: 4–7 points Group III: 8–15 points

This final SPI-I did well in both Yale cohorts (outcome rates of 3, 27, and 48% *P* < 0.001) and Canadian cohorts (outcome rates of 10, 21, and 59%, *P* < 0.001) in the three groups, although the outcome rate in risk group I of Canadian cohort remained high. The difference in performance in the two cohorts was attributed to the probable inaccuracy in classifying coronary heart disease in the Canadian cohort (3).

SPI-I provided a convenient prognostic scoring system using clinical parameters which were readily available. The outcome rates among the three risk groups were large, and it was reproduced in an independent cohort (3). Despite this, the developers of SPI-I suggested that more studies were needed to confirm that the weights assigned to the risk factors of SPI-I were accurate. This eventually led to the creation of SPI-II. Also, stroke alone was not considered in SPI-I because excluding early mortality could lead to false stroke risk outcome. It also did not distinguish between ischemic stroke and hemorrhagic stroke in the stroke outcome. Further validation of SPI-I came in 4 independent cohorts during the development of SPI-II. SPI-I performed fairly in these validation cohorts, but SPI-II outperformed SPI-I (4).

SPI-II is a revised version of SPI-I that was introduced and internally validated by the same investigators in 2000. SPI-II was intended to predict the risk of stroke or death within 2 years of TIA or ischemic stroke. This clinical score included two more risk factors—congestive heart failure and prior stroke. The points assigned to different risk factors were also modified in SPI-II. The risk factors of SPI-II and allocated points are detailed in Table 2. This modified risk score is the result of the work done to validate and improve SPI-I in 4 test cohorts. These cohorts were from Women's Estrogen for Stroke Trial (WEST), United Kingdom Transient Ischemic Attack (UK-TIA) Aspirin Trial, the Clopidogrel vs. Aspirin in Patients at Risk of Ischemic Events (CAPRIE) Trial and the Northern Manhattan Stroke Study (NoMaSS). Data from the WEST cohort was used to recognize new variables to develop SPI-II, and the other three cohorts were used to validate the score (4).

The pooled rates of stroke or death within 2 years (in the 3 cohorts excluding the cohort used to develop SPI-II) for SPI-I were 9, 17, and 23% in the three risk groups (Risk group I: 2,430 patients, II: 5,411 patients, III: 1,379 patients), respectively. For SPI-II, the pooled rates were 10, 19, and 31% in the three risk groups (Risk group I: 4,725 patients, II: 3,314 patients, III: 1,181 patients), respectively. In receiver operator characteristic (ROC) analysis, AUC for SPI-II was 0.63 (95% CI, 0.62–0.65) as compared to SPI-I whose AUC was 0.59 (95% CI, 0.57–0.60) verifying that SPI-II performed better than SPI-I. Nevertheless, the area under the curve of ROC analysis was only marginally better. In addition, it does not seem that SPI-II is performing better among patients in the lowest-risk group. Both SPI-I and II do not take stroke type and aortic plaque into account—two factors which might affect prognosis. Both SPI-I and II were developed and validated in research cohorts retrospectively, and their application in a non-research setting might differ. It should also be emphasized that the application of SPI scores are restricted to patients with carotid territory TIA or minor stroke.

In a large, prospective, community-based cohort of 5,575 patients, the C-statistic for SPI-II was 0.62 (95% CI, 0.61–0.64) (13). In another cohort of 1,897 patients with acute non-disabling ischemic stroke or TIA from German stroke centers with a median follow up of 1 year (14), SPI-II performed marginally better (recurrent stroke AUC 0.65 [95%CI, 0.6–0.7]; recurrent stroke or cardiovascular death AUC 0.66 [95% CI, 0.61–0.70]) than Essen Stroke Risk Score (ESRS), Hankey and Life-Long after Cerebral Ischemia (LiLAC) scores.

On the other hand, in a validation cohort of 592 patients with TIA or minor stroke, SPI-II overestimated the risk of outcome in low-risk patients and underestimated the risk in the high-risk group (discrimination C-statistics 0.64 [95% CI, 056–0.72) (15). In a population-based study in Oxfordshire, UK, SPI-II performed poorly in predicting the short-term risk of recurrent stroke (16), which is unsurprising because SPI-II was intended to predict long-term risk of stroke or death. Overall, SPI-II has fair AUROC/c-statistics (0.6–0.72) in these studies (13–15) for long-term risk prediction. But the SPI-II score is a poor model for predicting the short-term risk of stroke.

### Essen Stroke Risk Score (ESRS)

Essen Stroke Risk Score (ESRS) (5) was developed from the data of subgroup of stroke patients from the CAPRIE trial (17) to predict 1 year risk of recurrent stroke. The parameters of ESRS and points assigned to these parameters and the separation of patients into low- and high-risk groups are detailed in Table 3.

**Table 3:** Parameters in ESRS and in modified ESRS.

	**ESRS** **Points**	**Modified ESRS** **Points**
**Risk factors**
Age:		
<65 years	0	0
65–75 years	1	1
>75 years	2	2
Hypertension	1	1
Diabetes mellitus	1	1
Previous myocardial infarction	1	1
Other cardiovascular disease (except myocardial infarction and atrial fibrillation)	1	1
Peripheral artery disease	1	1
Smoking	1	1
Additional TIA or ischemic stroke in addition to a qualifying event	1	1
Stroke subtype except small artery occlusion	–	1
Waist circumference >= 90 cm	–	1
Male Gender	–	1
Risk groups	Low-risk group: (0–2 points), High-risk group: (≥3 points)	Men: Low-risk group: (0–5 points), High-risk group: (≥6 points) Women: Low-risk group: (0–4 points), High-risk group: (≥5 points)

In a validation cohort of 852 patients with acute ischemic stroke or TIA in German stroke units, patients with an ESRS >= 3 (compared to patients with ESRS <3) had a significantly higher risk of recurrent stroke or cardiovascular death (9.7% vs. 5.1%; OR 2.00, 95% CI, 1.08–3.70) (18). Further validation came from a large cohort of 15,605 outpatients with previous TIA or stroke from the Reduction of Atherosclerosis for Continued Health (REACH) registry (19), in which ESRS accurately stratified the risk of recurrent stroke or major vascular events. On the other hand, ESRS (and SPI-II) performed poorly in predicting short-term recurrent stroke risk in a population-based study in Oxfordshire, United Kingdom (16).

Performance of SPI-II (recurrent stroke AUC 0.65 [95% CI, 0.60–0.70]; combined recurrent stroke or cardiovascular death AUC 0.66 [95% CI, 0.61–0.70]) was marginally superior to ESRS (recurrent stroke AUC 0.62 [95% CI, 0.57–0.67]; combined recurrent stroke or cardiovascular death AUC 0.65 [95% CI, 0.60–0.69] as shown in a prospective study of German stroke center cohort (14) but ESRS has been validated in a stable outpatient population with cerebrovascular disease. In another study (20) of 730 patients with TIA or ischemic stroke, the AUC for ESRS was 0.59. In a Chinese cohort of 11,384 patients with TIA or ischemic stroke, ESRS and SPI-II had similar predictive value (21). However one study found ESRS had a superior AUC compared to SPI-II (0.677 vs. 0.553) and even concluded that ESRS might be suitable for short-term prediction of stroke despite a very small sample size (*n* = 167) (22). In a prospective Chinese cohort of 3,316 outpatients with ischemic stroke, ESRS had c-statistics of 0.63 (0.57–0.69) for recurrent stroke and 0.63 (0.58–0.68) for combined vascular events (23).

A modified ESRS was generated by adding scores for waist circumference, stroke subtype categorized by etiology, gender to the ESRS and it was validated using a large prospective cohort of 3,588 patients (EVEREST cohort) with ischemic stroke in Japan (24). The C-statistics for recurrent ischemic stroke was 0.632 (95% CI, 0.579–0.684) in the modified ESRS and was 0.604 (95% CI, 0.554–0.654) for the original ESRS. For cardiovascular events, c-statistics were 0.64 (95% CI, 0.590–0.661) in the modified ESRS and 0.613 (95% CI, 0.564–0.689) for ESRS. While modified ESRS showed better c-statistics, there were limitations to this study. The incidence of recurrent ischemic stroke was low (121 events in 3,292 patients) and it was not confirmed in a validation cohort although the EVEREST data was externally validated using a cross-validation method.

### Recurrence Risk Estimator at 90 days (RRE-90)

Recurrence Risk Estimator at 90 days (RRE-90) is a web-based prognostic scoring system designed to predict 90 day recurrent stroke risk in patients presenting with ischemic stroke. RRE-90 actually consists of 2 models- model A is a clinical-based model, whereas model B takes both clinical and imaging findings into account (6). The parameters included in RRE-90 were a history of TIA or stroke within the month preceding index stroke, admission stroke subtype according to Causative Classification of Stroke System (CCS), and MRI imaging findings—isolated cortical infarcts, multiple acute infarcts, simultaneous infarcts in different vascular territories, and multiple infarcts of different ages. Some of the vascular risk factors such as hypertension and diabetes, which have been associated with stroke and included in many other scoring systems have not been included in RRE-90.

The primary derivation cohort of RRE-90 consisted of 1,458 patients with ischemic stroke identified retrospectively. RRE-90 demonstrated good discrimination not only in the initial study (AUC: 0.7–0.8) but also in another cohort of 433 patients (AUC: 0.7–0.76) (6). Nevertheless, the retrospective design, incomplete follow-up (half of the patients completed the follow-up), and single hospital setting were some of the limitations of this study.

The validity of RRE-90 was tested in 1,468 patients with MRI-confirmed acute ischemic stroke from 3 hospital-based cohorts (the United States, Brazil, and South Korea) (25) and AUC ROC curve for discrimination was found to be 0.76 (95% CI, 0.70–0.82). In a retrospective study of 257 consecutive patients with DWI-positive TIA, the sensitivity and specificity of an RRE score of ≥2 for predicting 7 day stroke risk were 87 and 73%, respectively (AUC: 0.85, 95% CI, 0.78–0.92) (26). In a prospective study, c-statistic of RRE-90 for 90 days recurrence was 0.681 (95% CI, 0.592–0.771) (27). Both of these studies showed better discrimination for RRE-90 compared to the ABCD^2^ score. However, the study population was limited to patients with DWI-positive TIA or minor stroke. Compared to some other scoring systems including ABCD^2^ score, it seems that RRE-90 has a better predictive value for early recurrence risk after cerebral ischemia; however, it requires both accurate subtyping and neuroradiological assessment.

### Models Based on the Dutch TIA Trial (DTT) and Life-Long After Cerebral Ischemia Trial (LiLAC)

In the Dutch TIA Trial, 997 patients with TIA and 2,130 patients with minor stroke were enrolled for 2 year risk analysis for outcomes of fatal or non-fatal stroke in addition to myocardial infarction, stroke, or vascular death (7). It identified the following independent risk factors for stroke, myocardial infarction or vascular death: age > 65 years; male sex; dysarthria; multiple attacks; diabetes; angina pectoris; intermittent claudication; computed tomographic evidence of any cerebral infarct especially a border zone infarct or white matter hypodensity; and electrocardiographic evidence of anteroseptal infarct, ST depression, left ventricular hypertrophy, or left atrial conduction delay. Prognostic models derived from DTT showed poor calibration and discriminative value (28). In a validation cohort consisting of 592 patients with TIA or minor stroke, Dutch TIA model was found to overestimate the risk and discrimination was poor (c-statistic 0.64, 95% CI, 0.56–0.72) (15).

The Dutch TIA cohort was followed-up for a mean period of 10 years, and the data was reanalyzed in LiLAC (8). Three models were designed to predict the outcome of all-cause mortality and the composite of the event of death from all vascular causes, non-fatal stroke, and non-fatal myocardial infarction. The AUC-ROC for all the three models reached >0.8, but this could not be replicated in a validation cohort (14).

### Hankey Score

Hankey Score was derived from a cohort of 469 TIA patients without prior stroke and evaluated prospectively over an average period of 4.1 years (range 1–10 years). The major outcome events were a stroke, coronary event, stroke, MI, or vascular death. It used 8 prognostic factors to determine a 5 year risk percentage. These factors included age, gender, affected region (amaurosis fugax, carotid as well as vertebrobasilar TIAs) frequency of TIA, peripheral vascular disease, left ventricular hypertrophy, and residual neurological signs (9).

In two independent cohorts (1,653 TIA patients in UK-TIA aspirin trial and 107 TIA patients in Oxfordshire Community Stroke Project), the reliability of Hankey score was good for lower-risk patients, but it overestimated risk in the higher risk group (29). In a validation cohort of 592 patients with recent TIA or minor stroke (15), Hankey score overestimated the 2 year risk, and its discrimination c-statistics was found to be 0.63 (95% CI, 0.54–0.72). In the 2,381 patients with TIA or non-disabling ischemic stroke from German stroke centers (14), Hankey score had AUC 0.62 (95% CI, 0.57–0.67) for recurrent stroke and AUC of 0.64 (95% CI, 0.60–0.69) for the combined endpoint of stroke or cardiovascular death. Nevertheless, the clinical usefulness of this score is questionable because it involves a sophisticated equation to determine the 1 or 5 year stroke-free survival after TIA.

### California Risk Score and ABCD Score

California risk score was derived from a retrospective cohort of 1,707 patients identified by ED physicians as having TIA to predict the risk of stroke in the 90 days following TIA (10). This simple score was found to be useful in estimating the risk which varied from 0% in patients with score 0 to 34% in patients with score 5. The risk factors included in this score are detailed in Table 4.

**Table 4:** Clinical parameters in California Risk Score, ABCD and ABCD^2^.

	**California Risk Score**	**ABCD**	**ABCD^**2**^**
Age ≥ 60 years	1	1	1
Blood Pressure ≥ 140/90 mm Hg	–	1	1
Clinical features	Unilateral weakness: 1	Unilateral weakness: 2	Unilateral weakness: 2
	Speech impairment: 1	Speech disturbance without weakness: 1	Speech disturbance without weakness: 1
Duration of symptoms	>10 min: 1	≥60 min: 2 10–59 min: 1	≥60 min: 2 10–59 min: 1
Diabetes	1	–	1
	Maximum score: 5	Maximum score: 6 Intermediate to High-risk group: >3 Low-risk group: ≤3	Maximum score: 7 High-risk group: >5 Intermediate-risk group: 4–5 Low-risk group: <4

ABCD score (Table 4) was derived from a population-based cohort of patients (*n* = 209, Oxfordshire Community Stroke Project) with a probable or definite TIA to estimate the 7 day risk of stroke and it was validated in a similar cohort of 190 patients (Oxford Vascular Study; OXVASC cohort) (11).

There have been several studies into ABCD score—many of these studies (30–35) have validated the predictive value of ABCD in predicting short-term stroke risk; however, many of these studies have several limitations including retrospective design and small sample size. Several validation studies have concluded that ABCD failed to identify all the high-risk patients (36, 37) or some patients assigned to the low-risk group also had a high risk of cerebral ischemia or radiographic evidence of infarction despite transient symptoms (38).

The investigators who derived California risk score and ABCD score joined to validate the two scores in four validation cohorts (12) for stroke risks at 2, 7, and 90 days (c statistics 0.60–0.81). One of the aims of this study was to derive and validate a unified score from these scores. The resultant score ABCD^2^ had improved c-statistics in both the derivation cohorts and validated well (c-statistics 0.62–0.83).

In a study to compare ABCD, ABCD^2^ and California score and to evaluate their relationship to acute ischemic lesions (positive DWI), it was found that elevated California, ABCD or ABCD^2^ scores were not associated with positive DWI (39). In another study comparing the performance of these three scores, the c-statistics for high-stroke risk was not significantly different between the three scores (40).

### ABCD^2^ Score

ABCD^2^ is the unified score derived from original California and ABCD score to predict short-term (2, 7, and 90 days) risk of stroke among patients with TIA. The risk factors included in the ABCD^2^ score are detailed in Table 4.

ABCD^2^ score has been widely replicated and studied in different cohorts with conflicting results. While many studies (15, 16, 33, 35, 37, 40–53) have validated ABCD^2^ with c-statistics/area under the ROC curve ranging from 0.57 to 0.88 (not all of these studies provided c-statistics/AUC), there were a few studies which have raised doubts about the predictive value of ABCD^2^ (54–67). In particular, high rates of recurrence was observed in low-risk groups in studies with large sample size (54, 56, 57). In a study to assess the performance of ABCD^2^ score in TIA patients subcategorized as tissue-positive or tissue-negative on DWI or CT imaging, it was found that positive tissue patients with low ABCD^2^ and tissue negative patients with high ABCD^2^ had similar stroke risks. Another study found that 1 in 5 patients with low ABCD^2^ score had symptomatic vascular stenotic lesions (68). Use of ABCD^2^ by non-stroke specialists was not reliable to risk-stratify patients according to some studies (58, 69, 70).

### ABCD^2^ With Additional Variables

Apart from validating ABCD^2^, many of these studies were done in an attempt to improve the performance of ABCD^2^ by adding additional variables such as C-reactive protein (CRP), diffusion-weighted imaging (DWI), CT or carotid imaging (Table 5). For example, one study (72) showed that including CRP measurements in the acute phase to ABCD^2^ improved the AUC significantly, but the TIA patient sample size was small and lacked validation. Other studies have also shown that adding parameters like DWI findings alone (44), brain infarction on DWI or CT (46), carotid imaging (at least 50% stenosis) and abnormal DWI (48), etiology and DWI (43, 71, 73), CT or transcranial Doppler (74), or carotid artery intima-media thickness (75) to ABCD^2^ increase the c-statistics/AUC of ABCD^2^ significantly. But many of these predictive models lack validation in large size cohorts. ABCD^2^ with DWI seems to be the most predictive out of these models.

**Table 5:** Scoring systems derived from ABCD/ABCD^2^.

**Study**	**Additional variables**	**Study type**	**Study population**	**Index event**	**Outcome**	**Validation studies**
ABCD^2^ + MRI	DWI imaging lesion and vessel occlusion status	Prospective cohort (41)	180 patients	TIA or stroke	Recurrent stroke and functional impairment at 90 days	–
Clinical and Imaging-based model (CIP)	DWI	Prospective cohort (42)	601 patients	TIA	Stroke at 7 days	–
ABCD^2^-I	Brain infarction on imaging: either DWI (acute infarction) or CT (any infarction irrespective of presenting symptom)	A multicenter study with pooled analysis of unpublished data from 12 centers over 11 year period (46)	4,574 patients	TIA	Stroke at 7 and 90 days	2/2
ABCD^3^	Dual transient ischemic attack	Pooled international multicenter analysis of prospective cohorts (48)	2,654 patients	TIA	Stroke at 2, 7, 28, and 90 days	2/2
ABCD^3^-I	>= 50% stenosis on carotid imaging; Abnormal DWI	Pooled international multicenter analysis of prospective cohorts (48)	2,654 patients	TIA	Stroke at 2, 7, 28, and 90 days	6/6
ABCDE+	Etiology and DWI positivity	Prospective cohort (71)	248 patients	TIA	Stroke or recurrent TIA within 90 days	2/2

ABCD^2^ + MRI was created by adding diffusion-weighted lesion and vessel occlusion status to the ABCD^2^ score. In a prospective cohort of 180 patients with TIA or minor stroke (41) to predict recurrent stroke and functional impairment at 90 days, the area under the curve was higher for ABCD^2^+MRI (0.88 vs. 0.78, *P* = 0.01).

*Clinical- and Imaging-based prediction of stroke risk after TIA (C*IP Model) was created to improve the prediction for 7 day stroke risk after TIA by DWI imaging to ABCD^2^. In a cohort of 601 patients with TIA, adding imaging to ABCD^2^ increased the area under the curve to 0.81 (95% CI, 0.74–0.88) from 0.66 (95% CI, 0.57–0.76) for ABCD^2^ alone (42).

ABCD^2^-I was the result of an international multicenter collaborative study in which unpublished data of 4,574 patients with TIA was used to determine the optimal weighting of brain imaging in the ABCD^2^ score (46). The resultant score ABCD^2^-I showed improved predictive power with the weighting of 3 points which was given for infarction on CT or DWI. The area under the curve increased from 0.66 (0.53–0.78) for ABCD2 to 0.78 (0.72–0.85) for ABCD^2^-I. The shortcomings of this study include the data quality which was obtained from 12 different cohorts over an 11 year period in different countries and using different imaging modalities. Nevertheless, ABCD^2^-I performed better than ABCD^2^ in all the individual cohorts except one.

In a cohort (76) of 410 patients with TIA, ABCD^2^-I showed the improved area under the curve of 0.77 for ABCD^2^-I from 0.59 for ABCD^2^ but this study compared the 1 year risk of stroke, unlike the derivation study.

ABCD^3^ and ABCD^3^-I scores were developed by the same investigators with aim to improve ABCD^2^ based on preclinical information (ABCD^3^) and after initial investigations were completed (ABCD^3^-I). ABCD^3^ was derived from ABCD^2^ by assigning 2 points for dual TIA and ABCD^3^-I by assigning 2 points for at least 50% stenosis on carotid imaging and another 2 points for abnormal DWI (48). A total of 2,654 TIA patients were included in the derivation cohort. C-statistics for ABCD^3^ and ABCD^3^-I at 2, 7, 28, and 90 days were improved compared to ABCD^2^. In a validation cohort of 1,232 patients, ABCD^3^ and ABCD^3^-I predicted early stroke at 7, 28, and 90 days but the performance of ABCD^3^ was similar to ABCD^2^ in this validation cohort.

In a cohort of 693 patients with TIA, ABCD^3^ and ABCD^3^-I had improved c-statistics of 0.61 and 0.66, respectively, for short-term prediction of stroke compared with ABCD^2^ (77). In another cohort of 239 patients with TIA, c-statistics of ABCD^3^-I (0.825, 95% CI, 0.752–0.898) was superior than that of ABCD^2^ score (0.694, 95% CI, 0.601–0.786; *P* < 0.001) (78). Other studies (79–81) have provided further validation to ABCD^3^-I.

ABCDE + was derived from ABCD-score by adding variables etiology and DWI positivity to predict stroke or recurrent TIA within 90 days. In a cohort of 248 patients with TIA (71), ABCDE+ had superior predictive power as it had better area under the curve (0.67 95% CI, 0.55–0.75) compared to ABCD^2^ (0.48, 95% CI, 0.37–0.58) and ABCD score (0.50, 95% CI, 0.40–0.61; *P* = 0.07). ABCDE+ was validated in a cohort of 150 patients with TIA with the superior area under the curve for ABCDE+ compared with ABCD^2^ for predicting stroke (0.64 vs. 0.60) and for predicting death (0.62 vs. 0.56) (73).

When comparing the AUC/C-statistic for the different scores (Table 6), there is considerable overlap with no clear superiority shown by any of the scores. Scores developed to predict long-term risk of stroke have also been studied for short-term prediction of stroke risk and vice-versa as is evident in Table 6. ESRS and SPI-II did not perform well when studied for short-term prediction of stroke recurrence. This is not surprising as they were developed for long-term prediction of stroke recurrence.

**Table 6:** Range of C-statistic/AUC-ROC for risk of stroke recurrence of different scores in different studies.

**Score/Prediction model**	**Patient population**	**Prediction for**	**Number of cohorts studied**	**C-statistic/AUC-ROC (Range)**
**I. For short-term risk prediction (≤90 days)**				
ABCD	TIA	Ischemic stroke	12	0.53–0.95
ABCD^2^	TIA	Ischemic stroke	25	0.39–0.97
	Minor stroke	Ischemic stroke	4	0.53–0.74
	Transient symptoms with Infarction (TSI)	Ischemic stroke	2	0.45–0.69
ABCD^2^-I	TIA	Ischemic stroke	1	0.72–0.85
ABCD^3^	TIA	Ischemic stroke	2	0.61–0.79
ABCD^3^-I	TIA	Ischemic stroke	2	0.66–0.92
ABCDE+	TIA	Ischemic stroke or TIA	1	0.55–0.75
California	TIA	Ischemic stroke	6	0.51–0.85
Dutch TIA	TIA and amaurosis fugax	Fatal or non-fatal stroke	1	0.73
	TIA and amaurosis fugax	MI, stroke or vascular death	1	0.75
ESRS	TIA or minor stroke	Ischemic stroke	4	0.42–0.79
RRE-90	Ischemic stroke	Recurrent ischemic stroke	3	0.70–0.82
	TSI	Recurrent ischemic stroke	2	0.59–0.92
SPI-II	Minor stroke	Recurrent ischemic stroke	1	0.39–0.60
	TIA and minor stroke	Ischemic stoke	1	0.41–0.69
**II. For log-term risk prediction (>90 days)**				
ABCD	TIA	Ischemic stroke	1	0.57–0.67
ABCD^2^	TIA	Ischemic stroke	3	0.54–0.75
	TIA	Ischemic stroke or TIA	1	0.58–0.68
	TIA and minor stroke	Ischemic stroke	1	0.58–0.71
	TIA and ischemic stroke	Non-fatal stroke, MI or vascular death	1	0.57–0.70
ABCDE+	TIA	Ischemic stroke or TIA	1	0.53–0.76
Dutch TIA	TIA and ischemic stroke	Non-fatal stroke, MI or vascular death	1	0.56–0.72
ESRS	TIA and ischemic stroke	Ischemic stroke	2	0.57–0.67
	TIA and ischemic stroke	Stroke or cardiovascular death	1	0.60–0.69
	TIA and ischemic stroke	Non-fatal Ischemic stroke or MI	1	0.58
	Ischemic stroke	Recurrent ischemic stroke	1	0.55–0.65
	Ischemic stroke	Combined outcomes of fatal or non-fatal stroke, MI, non-fatal unstable angina, and cardiac death	1	0.56–0.66
Hankey	TIA and ischemic stroke	Ischemic stroke	1	0.57–0.67
	TIA and ischemic stroke	Stroke or cardiovascular death	1	0.60–0.69
	TIA and ischemic stroke	Non-fatal stroke, MI or vascular death	1	0.58–0.74
LiLAC	TIA and ischemic stroke	Ischemic stroke	1	0.59–0.69
	TIA and ischemic stroke	Stroke or cardiovascular death	1	0.61–0.70
Modified ESRS	Ischemic stroke	Recurrent ischemic stroke	1	0.58–0.68
	Ischemic stroke	Combined outcomes of fatal or non-fatal stroke, MI, non-fatal unstable angina, and cardiac death	1	0.59–0.69
SPI-I	TIA and ischemic stroke	Ischemic stroke or death	1	0.57–0.60
SPI-II	TIA and ischemic stroke	Ischemic stroke	2	0.58–0.70
	TIA and ischemic stroke	Ischemic stroke or death	1	0.62–0.65
	TIA and ischemic stroke	Stroke or cardiovascular death	1	0.61–0.70
	TIA and ischemic stroke	Non-fatal stroke, MI or vascular death	1	0.61–0.75
	Ischemic stroke	Ischemic stroke or death	1	0.61–0.64

## Discussion

Several risk score models have been developed to predict stroke recurrence. However, differences in the methodologies used in the derivation cohorts, population, and outcome make it difficult to compare these risk predictive models. For instance, some of these models have been developed solely from either TIA cohorts (like ABCD^2^ and Hankey score), or from stroke cohorts (ESRS, RRE-90) while others were developed from a combined cohort of TIA and stroke patients (SPI-I and -II, Dutch TIA score and LiLAC score). Most of the derivation/validation studies used time-based definition of TIA. In a study where the performance of ABCD^3^-I was assessed in both time and tissue-based definition of TIA, the performance measures were similar (81). A different study indicated that patients with transient symptoms and cerebral infarction are at higher risk of recurrence (26). In addition, different scoring systems have distinct application or utility. For example, RRE-90, ABCD^2^, and its derivatives were developed to predict short-term recurrence while scores like SPI-I and II, Hankey, ESRS, Dutch TIA, and LiLAC scores were developed to predict the long-term recurrence. The take home message is that discrimination is poor for the currently available risk prediction models. ABCD^2^ was derived from TIA cohort to predict short-term risk, making the comparison with scores like SPI-II and ESRS challenging. Furthermore, the number of patients in the derivation cohorts of some of these predictive models were limited (e.g., SPI-I was derived from sample size of 142 patients) and models like SPI-II and ESRS were derived from research cohorts and validations were also carried out in research cohorts.

In addition to these differences, some models (like Dutch TIA and LiLAC) attempted to improve the precision of the models by a) increasing the number of variables, or b) including imaging parameters (i.e., RRE-90). However, there is a tradeoff between improvement in precision and the ease of use in clinical settings by non-stroke specialists and general adoption and clinical value; for instance, complex models (such as Hankey score) might not be easily used and integrated into clinical practice. All the above issues highlight why the predictive value of existing models might differ significantly in non-research population.

Ischemic stroke and its recurrence are multifactorial conditions with a combination of genetic, environmental, and vascular factors at play. While a number of risk factors are associated with increased risk of recurrent stroke, there are recurrences which cannot be explained by conventional risk factors (82, 83). In addition, early stroke risk factors might be different from long-term risk factors. Therefore, one prediction model will unlikely be able to be optimized for both short and long-term recurrence for TIA and stroke patients. All the stroke recurrence prediction models reviewed in this work have their own strengths, scopes and limits. It should be noted that recent advances in the management of TIA and minor stroke might also have influenced stroke recurrence rate and the diagnostic performance of current predictive models. Currently, The ABCD^2^ (especially with DWI) and RRE-90 are the two optimal models for the prediction of short-term risk of recurrent stroke whereas ESRS and SPI-II are the better options for the prediction of long-term risk.

Finally, to address some of the challenges of existing models, it might be valuable to consider addition of insight from biomarkers such as copeptin (84–86) and serum asymmetric dimethylarginine (ADMA) (87), cystatin C (88), Lipoprotein (a) (89), all of which have shown some level of association with recurrent stroke.

Alternatively, application of machine learning methods in combination with rich longitudinal data at various level of resolution could drive the next generation of prediction models that are better tailored to individual patients (90). While machine learning methods are relatively new for healthcare applications, there is already evidence to suggest these methods can outperform currently available tools. These have been used for prediction of outcomes in acute stroke (91) with better area under the curve for deep neural network model. Deep neural networks have been studied for ischemic stroke risk assessment with promising results (92). Artificial neural network have been used to differentiate stroke from stroke mimics (93) and to identify patients at high risk for TIA or minor stroke (94). The use of machine learning in prediction modeling is encouraging but require further studies.

Currently, electronic health record data can capture clinical data from patients and can be mapped with geocoded information from CENSUS and other databases with information about food and environment and, be enriched with genetic data whenever available. Such wealth of information can be used to extract insight and identify potential risk factors and be used in building more stable and generalizable predictive models that can seamlessly work in the background as part of the electronic health record system and provide decision support system on-demand. Advances in machine learning and artificial intelligence will be an important component of the healthcare system, but their integration and adoption still require an ideological shift among healthcare providers.

## Author Contributions

DC and RZ conducted the literature search, reviewed the included studies, and evaluated the validation of the models. DC wrote the first draft of the manuscript. VA and RZ wrote different sections of the manuscripts. VA, JL, CS, CG, and RZ contributed to significant editing and revisions of the manuscript. All authors read and approved the final version.

### Conflict of Interest

The authors declare that the research was conducted in the absence of any commercial or financial relationships that could be construed as a potential conflict of interest.
